# Exploring the Prevalence of Dry Eye Disease and Its Impact on Quality of Life in Saudi Adults: A Cross-Sectional Investigation

**DOI:** 10.3390/medicina61060976

**Published:** 2025-05-25

**Authors:** Mohammad A. Jareebi, Ayman A. Akkur, Dhiyaa A. H. Otayf, Ahmed Y. Najmi, Osama A. Mobarki, Eyad Z. Omar, Mohammed A. Najmi, Ali Y. Madkhali, Nasser A. N. Abu Alzawayid, Yasir M. Darbeshi, Abdulaziz Ali Alagsam, Ali Alabbas Ahmad Alhazmi, Omar Essa Mohammed Kirat, Ahmad Y. Alqassim

**Affiliations:** 1 Department of Family and Community Medicine, Jazan University, Jazan 88723, Saudi Arabia; aalqassim@jazanu.edu.sa; 2 Faculty of Medicine, Jazan University, Jazan 45142, Saudi Arabia; aymen.akour@gmail.com (A.A.A.); dhiyaaot@gmail.com (D.A.H.O.); ahmednajmiofficial@gmail.com (A.Y.N.); osmobarki@gmail.com (O.A.M.); eyadzaki42@gmail.com (E.Z.O.); m.najmim@gmail.com (M.A.N.); ali27yahyamad@gmail.com (A.Y.M.); n.azawayid@gmail.com (N.A.N.A.A.); yasseraldrbshy@gmail.com (Y.M.D.); 3 Prince Mohammed bin Nasser Hospital, Jazan 82943, Saudi Arabia; aaalagsam@moh.gov.sa (A.A.A.); alhazmi1426@gmail.com (A.A.A.A.); okirat@moh.gov.sa (O.E.M.K.)

**Keywords:** dry eye diseases (DED), quality of life, prevenance, Saudi Arabia

## Abstract

*Background and Objectives*: Dry eye disease (DED) is a multifactorial condition that affects quality of life (QoL). Symptoms like discomfort, blurred vision, and light sensitivity can negatively impact work efficiency, productivity, and psychological well-being. This study aimed to examine the relationship between DED and QoL, identify risk factors, and estimate DED prevalence in Saudi Arabia. *Materials and Methods*: This cross-sectional study included 1062 participants from Saudi Arabia, recruited via convenience sampling. Data were collected using an online questionnaire with three sections: sociodemographic information, the ocular surface disease index (OSDI) to assess DED severity, and the Arabic WHOQOL-BREF questionnaire to evaluate QoL. *Results*: Among participants, 77% suffered from DED. Males (β = −9.18, *p* < 0.001), postgraduate degree holders (β = −13.86, *p* = 0.001), and individuals with income >15,000 SR (β = −5.10, *p* = 0.023) had lower OSDI scores compared to reference groups (females, those with high school education or lower, and those with income <5000 SR, respectively), indicating a lower DED risk. Employed individuals, students (employed: β = 10.78, *p* < 0.020; students: β = 10.60, *p* < 0.016), divorced/widowed individuals (β = 18.70, *p* < 0.003), and those with diabetes, hypertension, and thyroid disorders showed higher OSDI scores. Higher OSDI scores correlated with lower QoL scores across all domains (physical: β = −0.26, *p* < 0.001; psychological: β = −0.22, *p* < 0.001; social: β = −0.25, *p* < 0.001; environmental: β = −0.20, *p* < 0.001). *Conclusions*: DED significantly affects all QoL domains. Risk factors include occupation, diabetes, hypertension, and thyroid disorders. Awareness and prevention efforts should be prioritized by institutions, while physicians should screen for DED in patients with chronic conditions. Further research is needed on the long-term effects of these risk factors and to improve management strategies.

## 1. Introduction

Dry eye disease (DED) is a multifactorial condition characterized by a persistently unstable or insufficient tear film that causes discomfort, visual impairment, ocular surface epitheliopathy, inflammation, and neurosensory abnormalities. The global prevalence of DED varies widely, ranging from 5% to 50%, as reported by studies conducted in the United States, Australia, and several countries in Asia [1,2]. In Saudi Arabia, two published studies reported prevalence rates of 49.5% and 74.9%, respectively [3,4].

Environmental factors, including climate conditions, air pollution, and digital device use, also contribute significantly to DED development. A comprehensive review by demonstrated that temperature, humidity, and air pollutants significantly impact tear film stability and ocular surface health globally [5]. Low relative humidity has been consistently associated with increased tear evaporation, decreased tear stability, and worsened DED symptoms in controlled studies [6]. Additionally, the widespread use of digital devices has emerged as a significant risk factor, with a recent systematic review and dose-response meta-analysis by Ha et al. reporting that each additional hour of daily digital screen time was associated with 21% higher odds of myopia, with this relationship following a sigmoidal pattern that suggests potential harm beyond one hour of daily exposure [7].

Several factors influence the development of DED, including systemic disorders, environmental and sociodemographic factors, and iatrogenic causes such as medications or surgical procedures [8,9,10]. DED presents with symptoms ranging from ocular discomfort, a key indicator of disease progression and treatment response, to unstable tear films, epitheliopathy, corneal disease, and conjunctival disease. These symptoms can eventually lead to visual and functional impairment [11]. In severe cases, DED can result in ocular pathologies such as ulceration, scarring, infectious keratitis, and ultimately, blindness [10].

Considering the relatively high prevalence of DED, studies have indicated that dry eye disease (DED) has a significant influence on the quality of life [12]. According to the World Health Organization (WHO), quality of life is defined as an individual’s subjective evaluation of their standing in life, considering the cultural and value systems of their environment. It encompasses their aspirations, expectations, norms, and worries, all within the context of their goals and values [13]. Many treatment approaches for DED require long-term management, often integrating dry eye treatment regimens into daily routines, which can burden individuals affected by the disease [12].

Symptoms of DED, including discomfort, blurred vision, and sensitivity to light, have been shown to reduce work efficiency and productivity [14]. Furthermore, DED is associated with psychological stress, anxiety, and depression, with many affected individuals having a history of seeking psychological counseling [15]. In light of the significant impact of DED on ocular health and quality of life, this study aimed to examine the relationship between dry eye disease and quality of life, identify influential factors, and estimate the prevalence of DED in Saudi Arabia.

## 2. Materials and Methods

### 2.1. Study Design and Setting

This cross-sectional study explored the prevalence of dry eye disease (DED), its determinants, and its impact on quality of life (QoL) among adults in Saudi Arabia. Cross-sectional designs are particularly well-suited for capturing prevalence and associated risk factors at a specific point in time, allowing for an efficient assessment of population health behaviors and outcomes [16]. Data collection encompassed multiple variables relevant to the population of Saudi Arabia, offering valuable insights about DED prevalence and characteristics in the nation.

### 2.2. Data Collection Tools

The data collection tool was developed through a review of validated instruments designed to measure dry eye disease (DED) and quality of life (QoL). Experts in ophthalmology and public health were consulted to ensure the questionnaire’s relevance and cultural suitability for the Saudi Arabian context. The final instrument comprised 49 questions, structured into three main sections: demographics, the ocular surface disease index (OSDI), and the Arabic version of the World Health Organization Quality of Life (WHOQOL-BREF).

The first section focused on demographic data, covering variables such as age, gender, employment status, education level, monthly income, residency, and geographical area. The second section was the OSDI—an established measure for assessing DED severity—containing 12 questions administered in Arabic. Participants’ responses were scored from 0 to 100 and classified as normal (0–12), mild (13–22), moderate (23–32), or severe (33–100) [17,18]. The third section incorporated the Arabic version of the WHOQOL-BREF questionnaire, which has 26 items to evaluate physical, psychological, social, and environmental dimensions of QoL. Higher scores indicate a better quality of life, and conversely, lower scores indicate a poorer quality of life [19,20].

### 2.3. Data Collection Process

Data collection was conducted entirely online from June to August 2024. During this period, the research team distributed the questionnaires through widely used social media platforms (WhatsApp, Twitter, Telegram, and others). Although no formal training was required for data collectors, the study team received internal briefings to maintain a consistent approach in addressing participant inquiries and clarifying any survey items. Quality control measures were upheld by utilizing validated questionnaires—namely; the ocular surface disease index (OSDI) and the WHOQOL-BREF—for assessing dry eye disease (DED) severity and quality of life (QoL); respectively. Additionally, routine checks were performed to identify and resolve duplicate or incomplete submissions in a timely manner.

Convenience sampling was utilized to recruit adults (18+) residing in Saudi Arabia who signed the informed consent without further restrictions. Individuals who did not meet these criteria (e.g., minors or non-residents) were excluded. As with many survey-based studies on health conditions, participants experiencing DED symptoms may have been more inclined to respond to the survey due to personal relevance, potentially influencing prevalence estimates. This common phenomenon in cross-sectional surveys was taken into account during data interpretation. Participants received clear instructions and could reach out for clarification at any time, ensuring accurate responses and a reliable dataset. This online approach capitalized on the widespread use of social media in Saudi Arabia, enabling a broad coverage of diverse demographic groups while minimizing barriers to participation.

### 2.4. Study Size

Sample size estimation for the current cross-sectional study was based on the following statistical formula:

$$n_{h}=\frac{\left(deff\right)\;\left(Z^{2}\right)\;\left(P\right)\;\left(1-P\right)\;\left(k\right)}{\left(d^{2}\right)},$$

where:

$n_{h}$ is the sample size in terms of the number of participants to be selected;

deff is the sample design effect;

Z is the statistic that defines the desired level of confidence;

P is an estimate of the key indicator (in this case, dry eye disease prevalence) to be measured by the survey;

k is a multiplier to account for the anticipated rate of non-response;

d is the margin of error to be attained.

In this study, a total of 1062 participants was determined based on the following assumptions. First, the Z-statistic of 1.96 corresponded to a 95% level of confidence. The design effect (deff) was set at 2 due to the absence of empirical data suggesting an alternative value. A 20% proportion was applied as the non-response multiplier (k). The level of P was set at 50% to ensure the largest possible sample size given the unknown prevalence of dry eye disease (DED). Lastly, a margin of error (d) of 5% was adopted. Although the questionnaire was distributed online through convenience sampling, these parameters served as a methodological guide to capture a representative snapshot of DED prevalence and its impact on quality of life (QoL) among adults in Saudi Arabia.

### 2.5. Data Analysis

Statistical data analysis was conducted using RStudio (version 4.2.3, R Foundation for Statistical Computing, Vienna, Austria). The main variable in this study was the OSDI score, which was analyzed to determine its relationship with various predictors and the outcome of interest. The predictor variables were divided into two main categories: (1) sociodemographic characteristics, including age, gender, nationality, education level, income, occupation, and marital status; and (2) health-related parameters, such as body mass index (BMI), smoking status, and chronic conditions (diabetes, hypertension, thyroid disease, and dyslipidemia). The outcome variables of interest included the four domains of quality of life (QoL)—physical; psychological; social; and environmental health—assessed using the WHOQOL-BREF questionnaire.

Our analysis began with an overview of the sample characteristics. Categorical variables were described using frequencies and percentages, while continuous variables were summarized with mean values and standard deviations (SDs). The normality of continuous data were evaluated using the Shapiro–Wilk test and visual inspection of Q-Q plots. Multiple linear regression models were then employed to explore the adjusted relationships between dry eye disease (DED) scores and the QoL domains, taking into account the identified predictor variables. The associations were quantified using regression coefficients (β), 95% confidence intervals (CI), and *p*-values. Statistical significance was determined with a *p*-value threshold below 0.05.

## 3. Results

### 3.1. Sociodemographic Characteristics of Participants

This study included 1062 participants, 56% of whom were females, with a mean age of 27 ± 10 years. The majority of the sample were Saudis (97%), students (60%), and singles (71%), with 74% having completed or currently pursuing a bachelor’s degree. Most participants earned a monthly income of less than 5000 riyals (34%) and resided in rural areas (57%) with a plain topography (40%). The remaining sociodemographic data are displayed in Table 1.

### 3.2. Habitual and Health-Related Characteristics of Participants

Table 2 shows the habitual and health-related characteristics of the sample. Regarding smoking, 85% of the participants had never smoked, while the remaining 10% and 5% were smokers and ex-smokers, respectively. When asked about specific forms of smoking, 5% reported smoking vape, 5% reported smoking cigarettes, and 6% reported smoking hookah and flavored tobacco. Among hookah and flavored tobacco users (*n* = 65), consumption was primarily occasional, with 38% using less than once weekly, 14% using once weekly, 12% using two to three times weekly, 15% using four to five times weekly, and 21% using daily. Among smokers (either current or ex-smokers), the mean number of cigarettes smoked per day was 6.1 ± 3.6 cigarettes. The prevalence of chronic diseases among participants was as follows: asthma (9%), hypertension (6%), thyroid disorders (5%), and sickle cell anemia (4%).

### 3.3. DED-Related Variables Characteristics of Participants (Based on OSDI Score)

**Figure 1:**
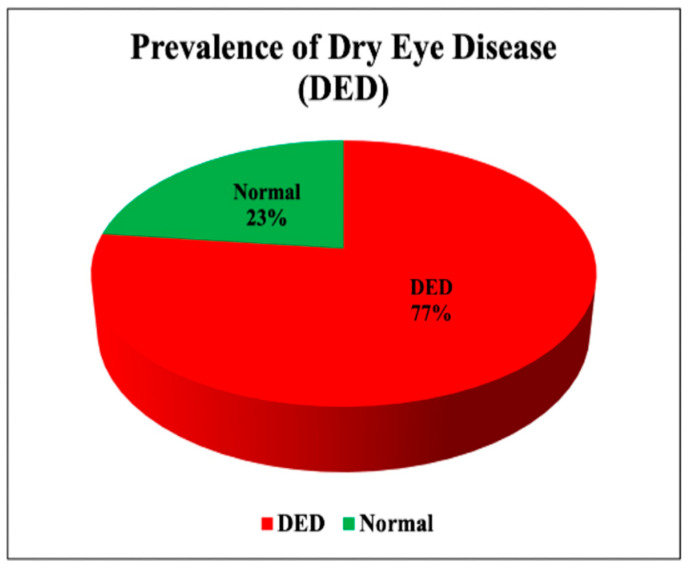
The prevalence of DED in the sample.

**Figure 2:**
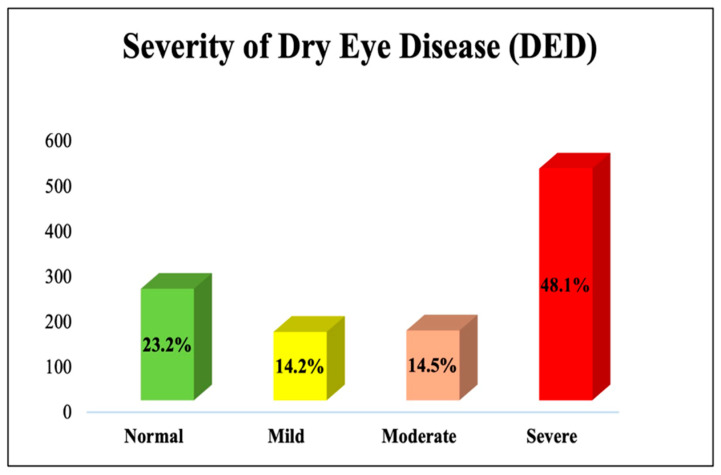
Distribution of dry eye disease (DED) severity levels in the sample.

The OSDI measure was used to assess DED severity among study participants, as presented in Table 3. DED severity was assessed solely through participants’ self-reported symptoms using the validated OSDI questionnaire [14,15]. Among the participants, the majority (77%) reported DED symptoms, as visually demonstrated in Figure 1. The OSDI scores of the participants ranged from 0 to 100, with a mean score of 36.46 ± 27.57. A higher OSDI score indicates a greater risk of DED. Based on the OSDI scores, the ocular surface health among the participants was categorized as follows: 23% had normal OSDI scores (no DED), 14% had mild DED, 15% had moderate DED, and 48% had severe DED. The DED severity categorization is clearly depicted in Figure 2.

### 3.4. Quality of Life Domains Among Participants

The overall QoL score of the participants, as presented in Table 4, ranged from 0 to 100, with a mean score of 72 ± 21. A higher score indicates a good QoL, and vice versa. The participants’ scores in the physical health domain averaged 65 ± 18, while the psychological health domain had a mean of 63 ± 20, the social health domain was 63 ± 25, and the environmental health domain was 63 ± 20. All QoL domains were measured using the validated Arabic WHOQOL-BREF questionnaire with scores transformed to a 0–100 scale according to the standard WHOQOL-BREF scoring protocol [16,17]. This transformation allows for standardized comparison across domains, with higher scores consistently indicating better quality of life.

### 3.5. The Relationship Between Study Variables and OSDI Score

Multiple linear regression analysis, displayed in Table 5, identified several significant predictors of OSDI score. Occupation had a significant impact, with employed and students having higher OSDI scores compared to unemployed individuals (Employed: β = 10.78, 95% CI: 1.69 to 19.86, *p* < 0.020; Students: β = 10.60, 95% CI: 1.96 to 19.24, *p* < 0.016). Divorced or widowed individuals had significantly higher OSDI scores compared to singles (β = 18.70, 95% CI: 6.52 to 30.89, *p* < 0.003). Additionally, a history of diabetes mellitus (DM), hypertension (HTN), and thyroid disease were all associated with higher OSDI scores (DM: β = 10.16, 95% CI: 0.93 to 19.39, *p* < 0.031; HTN: β = 11.08, 95% CI: 3.34 to 18.82, *p* < 0.005; thyroid disease: β = 11.77, 95% CI: 3.72 to 19.82, *p* < 0.004). Males had significantly lower OSDI scores compared to females (β = −9.18, 95% CI: −12.79 to −5.58, *p* < 0.001). Postgraduates also had significantly lower OSDI scores compared to individuals with a high school education or lower (β = −13.86, 95% CI: −22.36 to −5.37, *p* < 0.001). Furthermore, higher income was associated with a lower OSDI score, with those earning more than 15,000 SR showing a significant reduction (β = −5.10, 95% CI: −9.49 to −0.72, *p* < 0.023).

### 3.6. The Relationship Between DED and Physical Health (QoL Domain)

Table 6 presents the results from a multiple linear regression analysis assessing the relationship between OSDI scores and physical health, a component of quality of life (QoL). The findings indicate that higher OSDI scores were significantly associated with lower physical health scores, suggesting that worsening DED correlates with a decline in physical health-related QoL (β = −0.26, 95% CI: −0.30 to −0.23, *p* < 0.001). Additionally, individuals suffering from dyslipidemia (β = −4.53, 95% CI: −8.62 to −0.45, *p* < 0.030) had significantly lower physical health scores compared to their respective reference groups. Conversely, male participants (β = 3.35, 95% CI: 1.15 to 5.55, *p* < 0.003) and those with an income exceeding 15,000 SR (β = 2.99, 95% CI: 0.32 to 5.66, *p* < 0.028) reported significantly higher physical health scores compared to females and individuals with an income below 5000 SR, respectively. Other variables, including age, nationality, education level, marital status, body mass index (BMI), smoking habits, and other chronic conditions such as diabetes, hypertension, and thyroid disease, did not exhibit significant associations with physical health QoL. The model accounted for 24.2% of the variance in physical health scores.

### 3.7. The Relationship Between DED and Psychological Health (QoL Domain)

Table 6 presents the results of the multiple linear regression model examining the association between the OSDI scores and psychological health, a domain of QoL. The analysis shows that higher DED scores were significantly related to lower psychological health scores (β = −0.22, 95% CI: −0.27 to −0.18, *p* < 0.001). In contrast, being married was positively associated with psychological health (β = 5.34, 95% CI: 1.11 to 9.57, *p* < 0.014), relative to being single. Other factors, such as age, gender, nationality, education level, income, BMI, smoking habits, diabetes, hypertension, and thyroid disease, did not show significant relationships with psychological health QoL. The model explained 14.5% of the variance in psychological health scores.

### 3.8. DED Among Other Factors Affecting Social Health (QoL Domain)

Table 6 summarizes the multiple linear regression results for the relationship between DED scores and social health, another aspect of QoL. The results indicate that higher OSDI scores were significantly associated with lower social health scores (β = −0.25, 95% CI: −0.31 to −0.20, *p* < 0.001). Moreover, higher BMI (β = −0.27, 95% CI: −0.51 to −0.03, *p* < 0.030) and cigarette smoking (β = −11.01, 95% CI: −20.34 to −1.68, *p* < 0.021) were both associated with significantly lower social health scores compared to non-smokers. In contrast, being married was significantly associated with higher social health scores (β = 9.89, 95% CI: 4.64 to 15.14, *p* < 0.001) compared to being single. No significant associations were found for age, gender, nationality, education level, income, occupation, smoking history, diabetes, hypertension, and thyroid disease. The model accounted for 13.7% of the variance in social health scores.

### 3.9. DED Among Other Factors Affecting Environmental Health (QoL Domain)

Table 6 presents the results of the multiple linear regression analysis exploring the association between OSDI scores and environmental health, a QoL domain. Higher OSDI scores were significantly related to lower environmental health scores (β = −0.20, 95% CI: −0.24 to −0.15, *p* < 0.001). Additionally, the presence of thyroid disease (β = −5.83, 95% CI: −11.60 to −0.07, *p* < 0.047) was associated with lower environmental health scores compared to individuals without thyroid disease. Conversely, participants with an income above 15,000 SR (β = 3.58, 95% CI: 0.45 to 6.71, *p* < 0.025) had significantly higher environmental health scores compared to those with incomes below 5000 SR. Other variables, including age, gender, nationality, education level, occupation, marital status, BMI, smoking history, diabetes, and hypertension, did not show significant associations with environmental health. The model explained 15.8% of the variance in environmental health scores.

### 3.10. Summary of the Impact of DED on QoL

**Figure 3:**
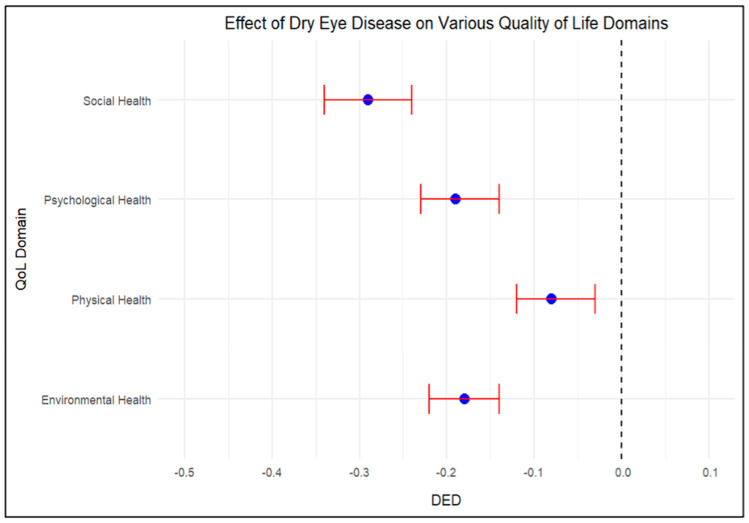
Effect of DED on Various Quality of Life Domains.

A summary of the findings (as shown in Figure 3) indicates that dry eye disease (DED) significantly impacts various quality of life (QoL) domains. The analysis revealed that a higher DED score is associated with lower QoL scores across all domains examined. Specifically, the physical health score showed the greatest reduction, with a beta coefficient of −0.26. Psychological health was notably affected, with a beta of −0.20. The social health score also showed a significant reduction, with a beta of −0.25. Lastly, environmental health also decreased significantly, with a beta of −0.20 (Figure 3).

## 4. Discussion

This study aimed to investigate the relationship between DED and QoL, identify contributing risk factors, and estimate the prevalence of DED in Saudi Arabia. Our results show that several factors are implicated in the development of DED. In this study, chronic diseases showed a significant correlation with DED. As mentioned, higher OSDI scores were found in those with a history of thyroid illness, hypertension, and diabetes mellitus (DM). These findings emphasize the necessity of considering ocular complications in the management plans of such patients.

Moreover, our findings also raise questions regarding the relationship between DED, QoL, and chronic illness. As seen, thyroid diseases can significantly impact patients’ QoL, raising concerns regarding the possible synergistic effect of DED and chronic diseases on QoL, regardless of whether DED was precipitated by the chronic disease.

Regarding occupation as a risk factor, our results show that students scored higher OSDI scores than those not working. These conclusions underline the critical nature of occupational status as a risk factor, which can be important to consider when assessing for DED.

Another noteworthy finding is the statistical significance of sex as a risk factor for DED, with men having significantly lower OSDI scores than women, indicating a lower risk for DED in males. Interestingly, our results also showed that men had higher physical health scores (QoL domain), further emphasizing our conclusions regarding the relation between DED and physical health.

These findings naturally lead to a discussion of QoL, as our results demonstrate a negative correlation between DED and QoL in all its domains. In Table 6, we notice that there is an increase in DED severity among participants with poorer physical health scores. DED may impair physical activity, potentially leading to reduced physical health. It is important to note that dyslipidemia was also associated with poorer physical health scores. With dyslipidemia being closely associated with physical activity, consideration for DED in such patients is vital.

DED severity was also negatively correlated with psychological health scores. The chronic nature of DED may play a significant role in its association with poorer psychological health, especially in the presence of other significant risk factors for poorer psychological health, like being single. DEDs negative impact on these two domains demonstrates the multidimensional nature of its impact on QoL.

Moreover, Table 6 demonstrates a strong association between higher DED scores and poorer social health, emphasizing the negative effects of DED on patients’ social functioning and relationships. Notably, higher BMI and cigarette smoking were linked to lower social health, suggesting that these factors may exacerbate the social difficulties faced by individuals with DED. In contrast, being married was associated with better social health, likely reflecting the protective role of social support in mitigating the disease’s adverse effects.

Similarly, Table 6 reveals a significant correlation between increasing DED severity and declines in environmental health, indicating that DED restricts individuals’ ability to interact with and adapt to their surroundings. These limitations may include difficulties in tolerating bright environments, outdoor activities, or exposure to dust and pollutants, all contributing to a decline in environmental health. The presence of thyroid disease was associated with even poorer environmental outcomes, potentially reflecting the combined effects of thyroid disorders and DED on patients’ ability to engage with their environment. Conversely, higher income appeared to mitigate the negative effects of DED, contributing to better environmental health, likely due to greater access to resources, healthcare, and treatment options that help address the environmental challenges posed by DED.

Figure 3 summarizes the overall impact of DED across all QoL domains, confirming that DED imposes a substantial burden on physical, psychological, social, and environmental well-being. The most pronounced decline was observed in physical health, followed by social, psychological, and environmental health, suggesting that the chronic discomfort and functional limitations associated with DED have far-reaching consequences across multiple aspects of life. These findings highlight the need to address not only the clinical management of DED but also its broader psychosocial and environmental impacts to improve patients’ overall quality of life.

Our finding of 77% DED prevalence is notably higher than global estimates, which typically range from 5 to 50% in general populations [1,2]. Several methodological factors may explain this discrepancy. First, online survey distribution may have introduced selection bias, as individuals experiencing DED symptoms might be more motivated to participate. This phenomenon has been observed in other online health surveys, as documented by Toscos et al. [21], who found that participants excluded from technology-based interventions due to lack of computer/internet access had worse health characteristics and more comorbidities. Second, our study was conducted during summer months in Saudi Arabia, when environmental factors such as higher temperatures, lower humidity, and increased air conditioning use may exacerbate DED symptoms. Van Setten et al. demonstrated that seasonal variation significantly impacts DED, with summer and winter being the two seasons most frequently associated with dry eye complaints (reported by 51% and 43% of patients, respectively) [22]. Their study of 738 patients across five European countries found that wind (71%), sunshine (60%), and heat (42%) were the most common weather conditions negatively impacting dry eye symptoms.

Furthermore, recent advancements in DED understanding have highlighted its multifactorial nature beyond traditional risk factors. Increasingly recognized contributors include microbiome dysregulation, with research showing that ocular surface inflammation may be triggered by changes in the ocular surface microbiota that influence immune responses [10]. Altered meibomian gland function has been established as the most common subtype of DED in both clinic and population-based studies, affecting the tear film lipid layer and contributing to tear film instability [23]. Additionally, neuroinflammatory mechanisms play a critical role in DED pathogenesis, involving bidirectional interactions between the nervous and immune systems, where nerve damage can trigger inflammatory responses while immune cells can affect nerve function through cytokine secretion [24]. Future studies should incorporate these emerging perspectives to better contextualize prevalence data

Looking at previously published studies, we can see that most of the conclusions of our study align with theirs. Regarding potential risk factors for DED, a 2022 cross-sectional Saudi study found that DED was more prevalent among females, which is in line with our conclusions. The study also emphasizes our conclusions on the relationship between DED and thyroid diseases, as they found that it increased the risk for DED. On the other hand, we can notice a drastic difference in the prevalence of severe DED, with only 1.7% of their participants having severe DED, in contrast to the significantly higher percentage of our study (48.1%), with both studies using the OSDI scale [25]. Another Saudi study investigating the prevalence risk factors of DED found that 41.69% is a somewhat similar result to that of our study; however, their conclusions regarding gender, thyroid disease, and diabetes mellitus contradict that of our study, as they found that these factors were not associated with DED. Furthermore, their results show that age significantly correlated with DED, contradicting our conclusions [26]. Another recent Saudi study with about 4066 participants found the prevalence of severe DED symptoms to be 16.4% and 14.7% of the participants reporting both severe symptoms of DED and a previous diagnosis of DED, contrasting with the 48.1% of our participants reporting severe symptoms. The same study also found that age and being a female correlated with DED, conflicting with our conclusions on age while supporting our conclusions on gender [4]. Overall, there are inconsistencies across Saudi studies regarding the role of age, diabetes mellitus, thyroid disease, and gender in DED in addition to the variability in the reported prevalence of severe symptoms. Similar inconsistencies are evident in international studies [27,28,29]. Regarding our conclusions on the relationship between DED and QoL, previous studies confirm the negative effect of DED on quality of life; however, no previous study has used the OSDI Questionnaire to assess the severity of DED [14,30].

The correlation between DED and chronic diseases can be explained by the systemic inflammatory processes associated with these conditions, which may exacerbate ocular dryness. The greater DED severity observed in men could be linked to differences in occupational exposure, lifestyle, or diagnostic biases. The significant negative impact of DED on QoL, particularly physical health, likely results from the discomfort and limitations it imposes on daily activities. The association between dyslipidemia and poorer physical health highlights the interplay between systemic health conditions and DED. Furthermore, the impact of occupation suggests a complex relationship between physical activity, environmental exposures, and DED symptoms.

The findings highlight the importance of considering DED in the management of chronic diseases and emphasize the need for more targeted interventions that account for both clinical and psychosocial factors. Healthcare providers should incorporate a comprehensive assessment of risk factors, including occupation, sociodemographic status, and comorbidities, when managing DED. Public health efforts should focus on raising awareness of DEDs impact on QoL and encourage lifestyle modifications, such as smoking cessation and weight management, to improve outcomes. Future studies should also investigate the correlation between DED severity classifications and the frequency of symptoms throughout the day, which would provide valuable insights for clinical management and patient education.

This study has several strengths, including a large sample size of 1062 participants and the use of validated tools to assess dry eye disease (DED) severity and quality of life (QoL). The comprehensive analysis of sociodemographic, occupational, and health-related factors provides valuable insights into the multifactorial nature of DED and its broad impact on physical, psychological, social, and environmental well-being. Notably, this is the first nationwide study to explore the association between DED and QoL, filling a critical gap in regional research. However, the cross-sectional design limits the ability to infer causality, and the convenience sampling method may introduce selection bias. Additionally, reliance on self-reported data may lead to recall and response biases. A key limitation was relying exclusively on self-reported symptoms without clinical examination to confirm DED diagnosis. Despite these limitations, this study offers a solid foundation for understanding the impact of DED on QoL and underscores the need for targeted public health interventions and further longitudinal research.

## 5. Conclusions

In conclusion, there is a prominent association between DED and all four domains of QoL: physical, psychological, social, and environmental. Certain significant risk factors for DED, such as occupation, should be taken into account by both corporations and educational institutions. These institutions should implement appropriate measures to raise awareness of DED and strategies for its prevention. Moreover, diabetes mellitus, hypertension, and thyroid disorders were identified as significant risk factors for DED. Consequently, physicians should routinely screen for DED in patients with these conditions and refer advanced cases to ophthalmologists. Additionally, environmental factors such as temperature, humidity, and digital device exposure play a critical role in DED management, requiring consideration in comprehensive treatment approaches. Future research should focus on the long-term impact of these risk factors on DED, strategies to improve the quality of life among DED patients, and further exploration of certain risk factors, such as DM and thyroid disorders, to better understand their relationship with DED.

## Figures and Tables

**Table 1:** Sociodemographic characteristics of study participants (*n* = 1062).

**Characteristics**	**Mean ± SD**
**Age**	27 ± 10 years
**Characteristics**	**Frequency (%)**
**Sex**	
Male	464 (44%)
Female	598 (56%)
**Nationality**	
Saudi	1029 (97%)
Non-Saudi	33 (3%)
**Education**	
High School or lower	225 (21%)
Bachelor	782 (74%)
Postgraduate	55 (5%)
**Occupation**	
Employed	279 (26%)
Student	636 (60%)
Unemployed	147 (14%)
**Marital status**	
Divorced/Widow	22 (2%)
Married	282 (27%)
Single	758 (71%)
**Income**	
“Less than 5000 SR”	364 (34%)
“5000–9999 SR”	207 (19%)
“10,000–14,999 SR”	212 (20%)
“≥15,000 SR”	279 (26%)
**Residence**	
Rural	607 (57%)
Urban	455 (43%)
**Topography**	
Coastal	428 (40%)
Mountainous	133 (13%)
Plain	501 (47%)

Abbreviations: SD: Standard deviation, n: Sample size, SR: Saudi Riyal (1 SAR ≈ 0.27 USD).

**Table 2:** Habitual and health-related characteristics of study participants (*n* = 1062).

**Characteristics**	**Frequency (%)**
**Smoking**	
Current	104 (10%)
Ex-Smoker	55 (5%)
Never	903 (85%)
**Cigarettes**	
No	1012 (95%)
Yes	50 (5%)
**Vape**	
No	1009 (95%)
Yes	53 (5%)
**Hookah and Flavored tobacco**	
No	997 (94%)
Yes	65 (6%)
**Smoking Per Week (** **SPW)**	
Less than once a week	25 (2%)
Once weekly	9 (1%)
Two to Three times a week	8 (1%)
Four to Five times a week	10 (1%)
Everyday	52 (5%)
**Characteristics**	**Mean ± SD**
**Cigarettes Per Day (CPD)**	6.1 ± 3.6
**Characteristics**	**Frequency (%)**
**Chronic diseases**	
Diabetes mellitus	46 (4%)
Hypertension	61 (6%)
Asthma	97 (9%)
Sickle cell anemia	40 (4%)
Thalassemia	15 (1%)
Thyroid disorders	53 (5%)

Abbreviations: SD: Standard deviation, n: Sample size.

**Table 3:** DED-related variable characteristics of study participants according to the OSDI (*n* = 1062).

Characteristics	Frequency (%)
**DED symptoms among participants**	816 (77%)
**Severity scale**	
Normal	246 (23%)
Mild	151 (14%)
Moderate	154 (15%)
Severe	511 (48%)

Abbreviations: SD: Standard deviation, n: Sample size, DED: Dry eye diseases.

**Table 4:** Quality of life (QoL) domains among the participants (*n* = 1062).

Characteristics	Mean ± SD
Overall score	72 ± 21
Physical health	65 ± 18
Psychological health	63 ± 20
Social health	63 ± 25
Environmental health	63 ± 20

Abbreviations: SD: Standard deviation, n: Sample size.

**Table 5:** Determinants of dry eye disease (DED) based on OSDI score.

Multiple Linear Regression			
	OSDI score		
Predictors	Beta	95% CI	*p*
Age	0.15	−0.16–0.45	0.342
Gender (reference: female)			
[Male]	−9.18	−12.79–−5.58	**<0.001** *
Nationality (reference: non-Saudi)			
[Saudi]	−6.99	−16.68–2.70	0.157
Education (reference: high school or lower)			
[Bachelor]	−6.99	−16.68–2.70	0.157
[Postgraduates]	−13.86	−22.36–−5.37	**0.001** *
Income (reference: less than 5000 SR)			
[Between 5000 and 9999 SR]	0.02	−4.67–4.72	0.993
[Between 10,000 and 15,000 SR]	−4.21	−8.98–0.56	0.083
[>15,000 SR]	−5.10	−9.49–−0.72	**0.023** *
Occupation (reference: unemployed)			
[Employed]	10.78	1.69–19.86	**0.020** *
[Student]	10.60	1.96–19.24	**0.016** *
Marital status (reference: single)			
[Married]	−3.56	−9.43–2.31	0.234
[Divorced/widow]	18.70	6.52–30.89	**0.003** *
BMI	−0.21	−0.47–0.06	0.132
Smoking history (reference: never)			
[Current]	5.67	−2.66–14.01	0.182
[Ex-smoker]	0.45	−7.20–8.10	0.909
DM (reference: no)			
[Yes]	10.16	0.93–19.39	**0.031** *
HTN (reference: no)			
[Yes]	11.08	3.34–18.82	**0.005** *
Thyroid disease (reference: no)			
[Yes]	11.77	3.72–19.82	**0.004** *
Observations	1062		
R2	0.14		

The remaining variables were tested but were statistically nonsignificant, so they were not included. Significant values are indicated in bold. * Significant, CI: Confidence interval, R2: Coefficient of determination, SR: Saudi Riyal (1 SAR ≈ 0.27 USD).

**Table 6:** Association between QoL Domains and Participants’ Characteristics.

Multiple Linear Regression (*n* = 1062)												
QoL Domain Scores	Physical Health			Psychological Health			Social Health			Environmental Health		
	*β*	*95% CI*	*p*	*Β*	*95% CI*	*p*	*Β*	*95% CI*	*p*	*Β*	*95% CI*	*p*
Severity of DED according to the OSDI score	−0.26	−0.30–−0.23	<0.001	−0.22	−0.27–−0.18	<0.001	−0.25	−0.31–−0.20	<0.001	−0.20	−0.24–−0.15	<0.001
Age	0.06	−0.12–0.25	0.512	0.11	−0.10–0.33	0.307	0.06	−0.21–0.33	0.653	−0.10	−0.31–0.12	0.372
Gender (reference: female)												
[Male]	3.35	1.15–5.55	0.003	0.66	−1.95–3.27	0.620	−0.97	−4.21–2.27	0.558	0.81	−1.77–3.38	0.539
Nationality (reference: non-Saudi)												
[Saudi]	2.34	−3.50–8.18	0.432	4.34	−2.59–11.27	0.219	0.95	−7.65–9.55	0.828	6.75	−0.08–13.59	0.053
Education (reference: high school or lower)												
[Bachelor]	−0.49	−2.99–2.02	0.703	−1.65	−4.62–1.32	0.275	−1.40	−5.09–2.28	0.456	−0.10	−3.03–2.83	0.944
[Postgraduates]	−2.01	−7.17–3.16	0.447	−4.82	−10.95–1.31	0.123	−0.21	−7.81–7.40	0.957	0.70	−5.35–6.75	0.821
Income (reference: less than 5000 SR)												
[Between 5000 and 9999 SR]	0.76	−2.08–3.60	0.599	−0.21	−3.58–3.16	0.903	−1.46	−5.64–2.72	0.493	1.12	−2.21–4.44	0.510
[Between 10,000 and 15,000 SR]	−0.37	−3.28–2.54	0.802	−0.74	−4.19–2.72	0.675	−3.21	−7.49–1.08	0.142	3.30	−0.10–6.71	0.057
[>15,000 SR]	2.99	0.32–5.66	0.028	1.32	−1.86–4.49	0.416	−0.67	−4.61–3.26	0.737	3.58	0.45–6.71	0.025
Occupation (reference: unemployed)												
[Employed]	5.52	−0.02–11.07	0.051	−2.36	−8.94–4.22	0.481	3.41	−4.75–11.57	0.412	1.62	−4.87–8.11	0.624
[Student]	4.99	−0.22–10.21	0.061	0.23	−5.95–6.42	0.941	3.10	−4.57–10.78	0.428	3.45	−2.65–9.55	0.268
Marital status (reference: single)												
[Married]	0.64	−2.93–4.21	0.726	−15.69	−29.86–−1.51	0.030	−14.66	−32.24–2.93	0.102	−7.66	−21.65–6.32	0.283
[Divorced/widow]	−3.48	−10.94–3.97	0.359	4.81	−1.01–10.63	0.105	−1.24	−8.46–5.98	0.737	1.60	−4.14–7.34	0.584
BMI	−0.02	−0.19–0.14	0.777	4.35	−0.96–9.66	0.108	0.32	−6.27–6.91	0.925	1.68	−3.57–6.92	0.531
Smoking history (reference: never)				7.36	−4.72–19.44	0.232	−5.71	−20.69–9.27	0.455	3.42	−8.49–15.34	0.573
[Current]	−1.61	−6.64–3.43	0.531	7.91	−1.85–17.67	0.112	−0.49	−12.60–11.62	0.937	2.06	−7.57–11.69	0.675
[Ex-smoker]	−0.96	−5.62–3.69	0.684									
Smoking Cigarettes (reference: no)				5.34	1.11–9.57	0.014	9.89	4.64–15.14	<0.001	3.21	−0.96–7.39	0.131
[Yes]	−0.88	−7.22–5.46	0.785	4.68	−4.16–13.53	0.299	2.29	−8.68–13.27	0.682	−0.97	−9.69–7.76	0.828
DM (reference: no)				−0.13	−0.33–0.06	0.185	−0.27	−0.51–−0.03	0.030	−0.02	−0.21–0.18	0.868
[Yes]	−0.62	−6.25–5.01	0.829									
HTN (reference: no)				0.36	−5.62–6.33	0.906	1.77	−5.64–9.18	0.640	−3.73	−9.62–2.17	0.215
[Yes]	−3.60	−8.32–1.12	0.135	−4.30	−9.82–1.23	0.127	−5.59	−12.44–1.26	0.110	−1.04	−6.49–4.41	0.708
Thyroid disease (reference: no)												
[Yes]	−2.24	−7.17–2.68	0.372	−3.83	−11.35–3.69	0.318	−11.01	−20.34–−1.68	0.021	−1.54	−8.96–5.88	0.684
Dyslipidemia (reference: no)												
[Yes]	−4.53	−8.62–−0.45	0.030	−0.64	−7.32–6.03	0.851	0.35	−7.93–8.63	0.934	−1.58	−8.17–5.00	0.637
R^2^ Value	0.242			0.145			0.137			0.158		

## Data Availability

The raw data supporting the conclusions of this article are available upon reasonable request to the corresponding author.

## Abbreviations

The following abbreviations are used in this manuscript:

DED	dry eye disease
QoL	quality of life
OSDI	Ocular Surface Disease Index
WHOQOL-BREF	World Health Organization Quality of Life–Brief version
